# Quercetin intake with exercise modulates lipoprotein metabolism and reduces atherosclerosis plaque formation

**DOI:** 10.1186/1550-2783-11-22

**Published:** 2014-05-27

**Authors:** Mahdi Garelnabi, Halleh Mahini, Thomas Wilson

**Affiliations:** 1 Department of Clinical Laboratory and Nutritional Sciences, University of Massachusetts, 3 Solomont Way, Suite 4 01854, Lowell, MA, USA

**Keywords:** Lipoproteins, Quercetin, Flavonoids, Inflammation, Exercise

## Abstract

**Study objectives:**

We proposed that mice supplemented with quercetin, a class of flavonoids known to have antioxidant and anti-inflammatory properties, will have profound effects on the pathophysiology of atherosclerosis when combined with exercise.

**Study design:**

Forty C57BL6 LDLr −/− mice were divided into four groups (n = 10): control untreated (NN); control group supplemented with 100 μg/day of quercetin (NQ); exercise group (EN); and exercise group supplemented with 100 μg/day of quercetin (EQ). All animals were fed atherogenic diet. The exercise groups were run on a treadmill for 30 minutes, 15 m/min for 5 days/week for 30 days. After 30 day animals were sacrificed and tissues were harvested.

**Results and conclusion:**

Mice supplemented with quercetin during exercise sessions had 78% atherosclerotic plaque reduction compared to control mice and 40% less atherosclerotic plaque formation compared to control group supplemented with quercetin. The manifestation of the combination of quercetin supplementation with exercise was more evident in the pro-reverse cholesterol transport genes, indicating a plausible mechanism for their combined beneficial effect.

The pathogenesis of atherosclerosis, the major cause of cardiovascular diseases (CVD), is multifactorial and therefore its treatment approaches and the ability to regress the plaque are complicated. Data from research on animal models and clinical studies have indicated that moderate daily exercise can alleviate the risk for the development of atherosclerotic plaques, while the same has not been true for the supplementation of antioxidants.

## Introduction

Atherosclerosis is a chronic disease of the large arteries and is a major cause of heart disease, stroke, and death in westernized societies. The etiology of cardiovascular disease (CVD) is complex and multifactorial, however there is substantial evidence [1,2] that oxidative stress [3] and inflammation [4] play an important role in the initiation and progression of the disease. Oxidative modification of low density lipoprotein (LDL) is believed to turn the otherwise native lipoprotein into an antigenic molecule that attracts monocytes turned macrophages to the vascular wall with a subsequent triggering of a complex immune response mediated by inflammatory modulators [5-7]. Recent insights into the pathogenesis of atherosclerosis underscore the importance of chronic inflammation in both the initiation and progression of the disease [8-11].

Exercise which induces a severe oxidative stress resulting in the depletion of plasma and tissue antioxidants has been shown to be an important deterrent of CVD [12-14]. This paradigm is supported by a large number of experimental animal studies and by epidemiological investigations. Over the past 5 decades, numerous scientific reports have examined the relationships between physical activity, physical fitness, and cardiovascular health [15-18]. Studies from our previous work have indicated that exercise induced the reverse cholesterol transport in mice that were exercised on a treadmill [19]. Others have reported that mice fed a high fat diet had increased numbers of macrophage clusters in adipose tissue [20], which were reduced by exercise training compared to sedentary mice. The sedentary mice also had higher levels of tumor necrosis factor α (TNF-α) mRNA, increased numbers of CD11c inflammatory macrophages and CD8 T cells [20]. Recently published study by Wen *et. al.*[21] reported that treadmill exercise training modulated hepatic cholesterol metabolism and circulating PCSK9 concentration in high-fat-fed mice.

Studies that combined antioxidants with exercise have also shown conflicting outcomes. Early study by Ramachandran et. al., [22] have showed that exercise reduced preexisting atherosclerotic lesions in LDL receptor knockout (−/−) mice, and that the addition of vitamin E supplementation to exercising did not reduce atherosclerotic lesion formation significantly when compared to untreated exercised mice [22]. Moreover, vitamin E supplementation was found to counteract the beneficial effects of exercise by preventing the induction of aortic catalase activity and endothelial NO synthase expression [23]. Flavonoids are a class of naturally occurring compounds widely present in fruits, vegetables and beverages derived from plants that have known antioxidant activity [24,25]. One such flavonoid, quercetin, has been shown to be an effective free-radical scavenger that inhibits lipoprotein oxidation [24]. Recent studies have also suggested that quercetin possesses anti-inflammatory properties as well as antioxidant activity. As an antioxidant and anti-inflammatory, quercetin appears to alleviate oxidative stress via diverse pathways, including NF-κB dependent mechanism [25], decrease activity of JAK3 [26], and/or by blocking the activation of pro-inflammatory/oxidative stress mediator signal transduction [27]. Quercetin has also been shown to prevent the accumulation of fat in the liver of mice fed a high fat diet [28] and to lower blood lipids in people with dyslipidemia [29]. Chang *et. al*. [30] have demonstrated that quercetin promotes cholesterol efflux from macrophages on a concentration-dependent manner through ATP-binding cassette transporter (ABCA-1) mediated mechanisms. It appears from these studies that the combination of exercise and quercetin supplementation may produce greater cardiovascular benefits than exercise alone. We propose that quercetin supplementation will have a profound effect on the pathophysiology of atherosclerosis when combined with exercise and that this action will be attributed to the inhibition of lipid oxidation, lowering of arterial lipid deposition and decreased development of plaque.

## Materials and methods

### Animals, diets, and exercise

All animal studies were performed in agreement with Public Health Service policy on use of laboratory animals, and in conformity with the Guide for the Care and Use of Laboratory Animals published by the US National Institutes of Health. The animal use protocol was approved by the Institutional Animal Care and Use Committee of the University of Massachusetts Lowell. All animals were fed an atherogenic diet containing 1.5% cholesterol as part of a 42% Fat Kcal Diet without antioxidants (Cat: TD.110489; Harlan Laboratories, Madison, WI).

Forty 4-week-old male LDLr^−/−m^ice on C57BL/6 J background (B6.129S7-Ldlr^tm1Her^/J strain) were obtained from Jackson Laboratory (Bar Harbor, ME). Mice were divided into four groups (10 mice each): control mice (NN) left untreated; control mice supplemented with quercetin (NQ); exercise group (EN); and exercise group supplemented with quercetin (EQ). Animals groups supplemented with quercetin were orally fed 100 μg/day, 5 days per week for 30 days 15 min prior to exercise. The quercetin solution was prepared in water with 1% sodium lauryl sulfate (SLS). Although the solution is very stable however; was gently mixed before pipetting to ensure correct dosage concentration. Pipette was used to deliver the correct amount; mouse was held upright until it swallowed the fluid. The exercise groups were run on a treadmill (Columbus Instruments, Columbus, OH) for 30 minutes at 15 meters/min, 5 days/week for 30 days.

### Materials

Quercetin was purchased from Cayman Chemicals (Ann Arbor, MI), with all other chemicals and reagents being purchased from Sigma-Aldrich Chemical Co. (St. Louis, MO). Gene expression reagents were obtained from Bio-Rad (Hercules, CA). Primers were designed and purchased along with TRIzol® from Life Technologies (Carlsbad, CA).

### Methods

Initially animals were acclimatized to the housing facility and the use of the treadmill instrument prior to starting the actual protocol. After 30 days of treatment the animals were fasted overnight (>12 hours), sacrificed with 100% CO_2_ exposure, and blood was collected via cardiac puncture. The plasma was collected after centrifugation at 4°C at 3000 rpm for 20 min and frozen at −80°C until assayed. The aorta and liver were perfused with cold phosphate buffered saline (PBS) prior to being harvested. All tissues were instantaneously frozen in liquid nitrogen following collection and stored at −80°C until assayed.

### Assessment of atherosclerotic lesions

At the completion of the livers perfusion and tissue collection the aorta was kept wet with cold PBS through the dissection process which was performed under a stereomicroscope from the iliac bifurcation up to the heart, including the beginning of the brachiocephalic, carotid, and subclavian arteries. Pictures of the aorta were obtained using a digital camera. Lesion area size was quantified using Image J software [31]. The lesion area was marked on the pictures under direct microscopic observation and quantified.

### Quantitative real-time PCR (qPCR)

Liver RNA was extracted using TRIzol according to the manufacturer’s protocol and the quantity was measured by Qubit (Life Technologies, Carlsbad, CA). cDNA was generated from 10–100 ng of total RNA and 1/20th of the sample was taken for qPCR. cDNA synthesis and qPCRs were performed with SYBR GreenER Two-Step qRT-PCR Kit according to the manufacturer’s protocol. qPCR was run in 20 μL of reaction mixture in sealed 96-well plates with iScriptTM Reverse Transcription Supermix and SsoFastTM EvaGreen® Supermix on an RTPCR MyiQTM2 system (Bio-Rad; Hercules, CA). Threshold cycle (CT) was determined by Bio-Rad iQ5 v.2.1 software. The melting curve and efficiency were assessed for all primer pairs. The level of mRNA was calculated using glyceraldehyde 3-phosphate dehydrogenase (GAPDH) as an internal control gene. Data are expressed as fold induction of mRNA level in one group compared to another.

### Enzyme-Linked Immunosorbent Assay (ELISA)

Plasma TNF-α, monocyte chemoattractant protein (MCP)-1, and interleukin (IL)-17α levels were determined according to manufacturer protocols by ELISA kits purchased from BioLegend (San Diego, CA).

### Statistical analysis

All data are presented as mean ± SD. Statistical significance for differences in lesion areas were evaluated using Student’s *t*-test. Statistical significance for relative gene expression was evaluated using one-way ANOVA with Tukey post-hoc test using 95% confidence intervals. Differences were considered significant at P <0.05.

## Results

All mice completed the study, tolerated the supplemented quercetin amount; there was no differences in the amount of consumed food between the groups or the physical appearance of the mice as a result of the quercetin intake. There was, however, a significant reduction in body weight in the EQ mice after 30 days of treatment compared to baseline (data not shown). The weight reduction appears to have resulted from the combination of the exercise and quercetin intake; however the mechanism for this weight loss is not very clear.

### Atherosclerotic lesion

**Figure 1:**
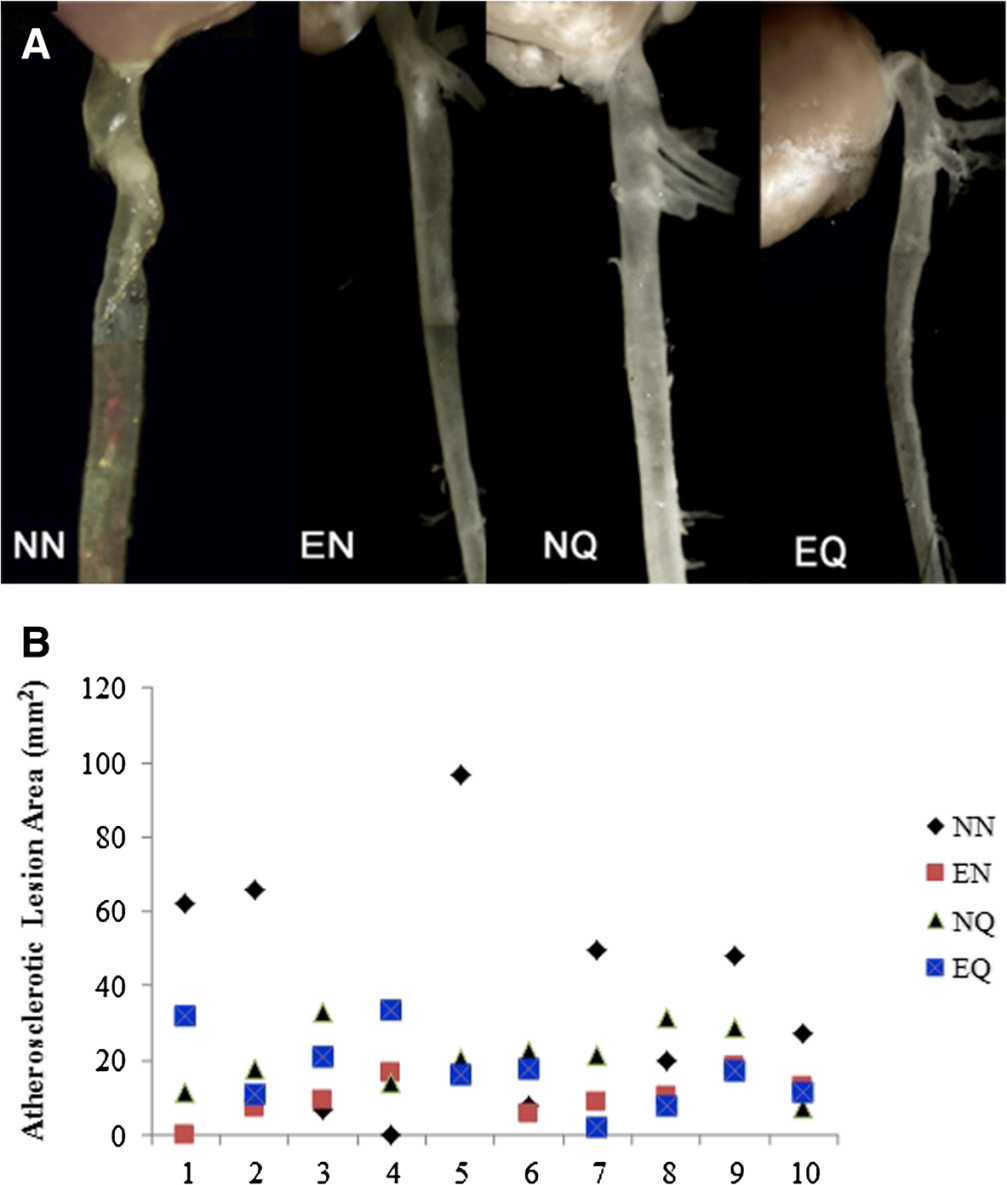
**Effect of quercetin and exercise on atherosclerotic lesion development. A**: Images of the atherosclerotic lesions in aortas. Atherosclerotic lesions in aortas of LDLr−/−mice fed a high-fat diet. NN: Control group; mice on atherogenic diet without quercetin and exercise treatment; EN: Mice on atherogenic diet and exercise without quercetin supplementation; NQ: Mice on atherogenic diet and quercetin supplementation; EQ: Mice on atherogenic diet, exercise and quercetin supplementation. Massive formation of atherosclerotic plaque can be seen on control and relatively less lesion formation on the other groups. **B**: Lesions areas dot plot representation in the 4 groups. EN: Mice on atherogenic diet and exercise without quercetin intake NQ: Mice on atherogenic diet and quercetin intake. EQ: Mice on atherogenic diet and exercise and quercetin intake. Compared to NN mice; the aorta lesion areas in EN, NQ and EQ showed significant decreases of 79%, 64% and 70% respectively (P < 0.05).

Atherosclerotic plaque formation in selected mice from all groups is shown in Figure 1A. The average lesion areas for the groups were: 56.04 mm^2^, 11.84 mm^2^, 19.95 mm^2^ and 16.63 mm^2^ for NN, EN, NQ, and EQ respectively, revealing a decrease of 79% (P < 0.01); 64% (P < 0.05) and 70% (P < 0.05) between each group, respectively, and the NN (Figure 1B).

### Plasma cytokines

**Figure 2:**
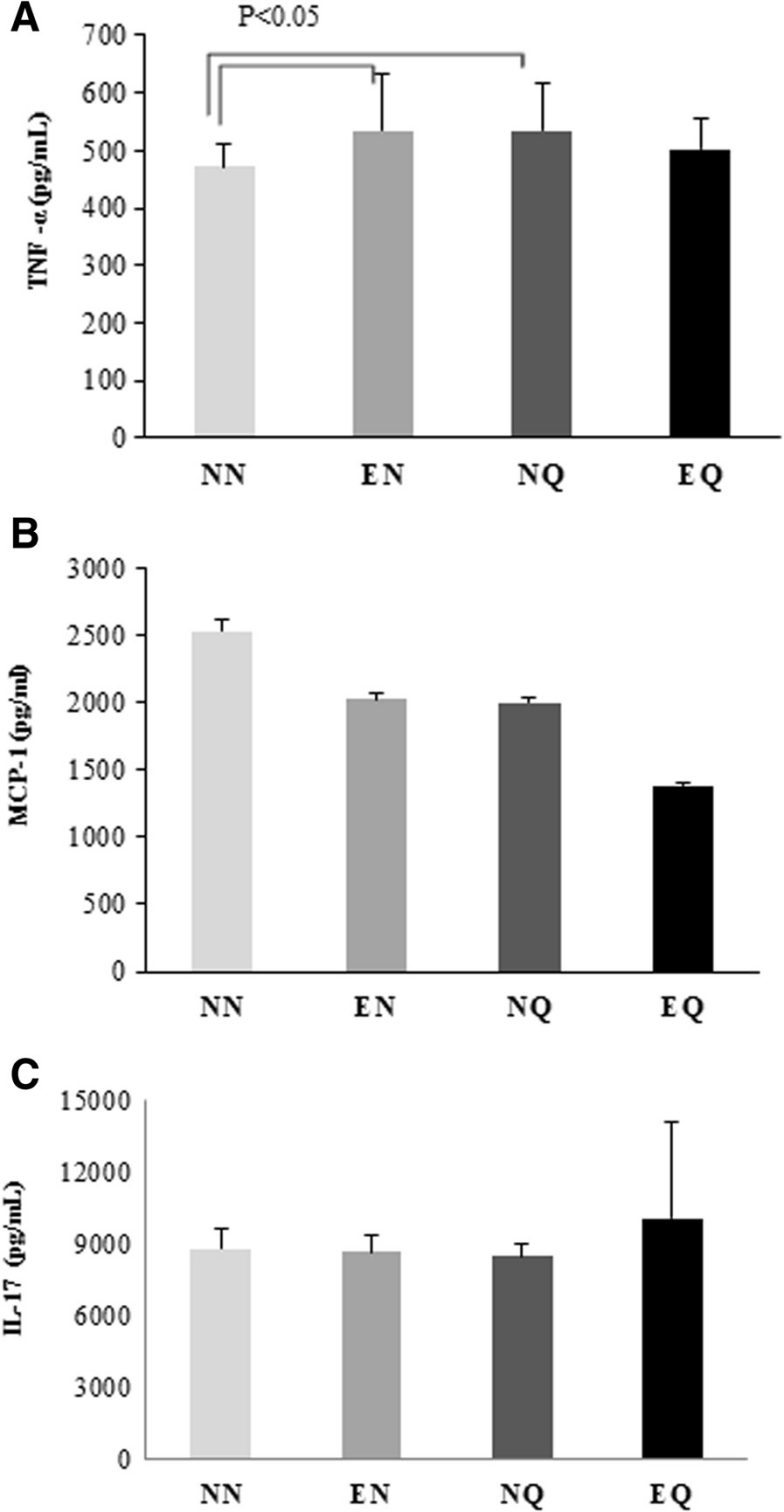
**Effect of quercetin intake and exercise on selected plasma biomarkers.** Plasma levels of TNF-α, MCP-1 and IL-17α. The figure shows average plasma levels of TNF-α **(A)**, MCP-1 **(B)** and IL-17 **(C)** . TNF-α levels significantly increased in the EN and NQ mice compared to NN group. However no significant changes were noticed between the groups MCP-1 and IL-17 levels.

The plasma concentrations of IL-17, MCP-1 and TNF-α measured by ELISA are shown in (Figure 2A,B and C). The average plasma concentrations for TNF-α were: 473.1 pg/mL, 534.4 pg/mL, 534 pg/mL and 502.3 pg/mL for the NN EN, NQ, and EQ groups respectively, depicting a significant increase (P < 0.05) in TNF-α level among the EN and NQ groups compared to the NN group.

On the other hand, plasma MCP-1 concentrations decreased among the EQ, EN, and NQ groups compared to the NN. The greatest decrease was observed in the EQ group (54.7%). The average plasma levels were: 2529.37 pg/mL, 2021.81 pg/mL, 1996.81 pg/mL, and 1384.69 pg/mL for the NN, EN, NQ, and EQ groups respectively.

The average plasma concentrations of IL-17 were 8775.0 pg/mL, 8646.6 pg/mL, 8460.6 pg/mL, and 10,053.1 pg/mL for the NN EN, NQ, and EQ groups respectively; showing an increase trend in the EQ group compared to the NN group but not significantly.

### Gene expressions in mouse liver

**Figure 3:**
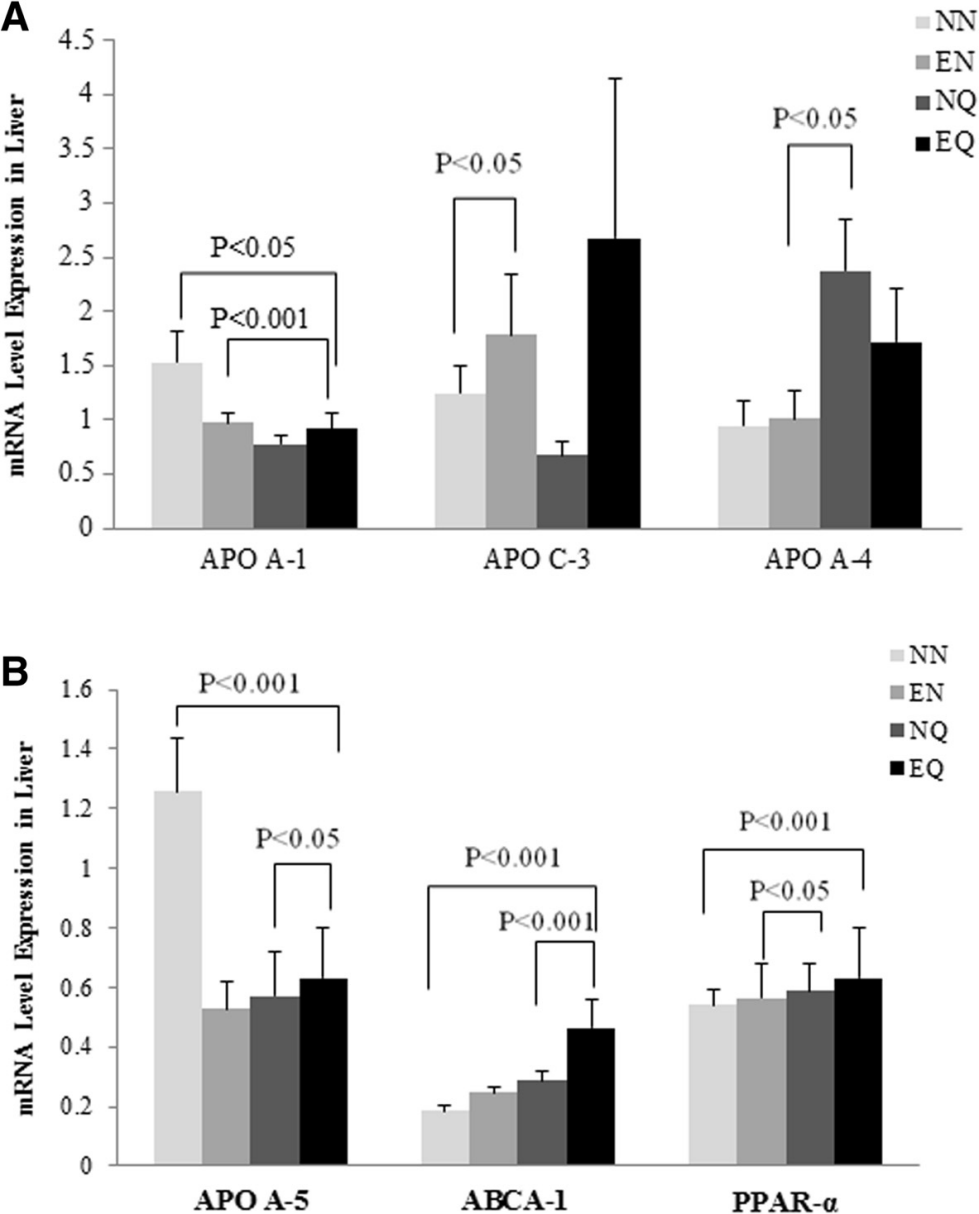
**Selected lipoproteins associated mRNA gene expression levels.** Levels of APOA-1, APOC3, APOA-4 mRNA expressions **(A)** and APOA-5, ABCA-1 and PPAR-α mRNA expression **(B)** are shown in these figures.

**Figure 4:**
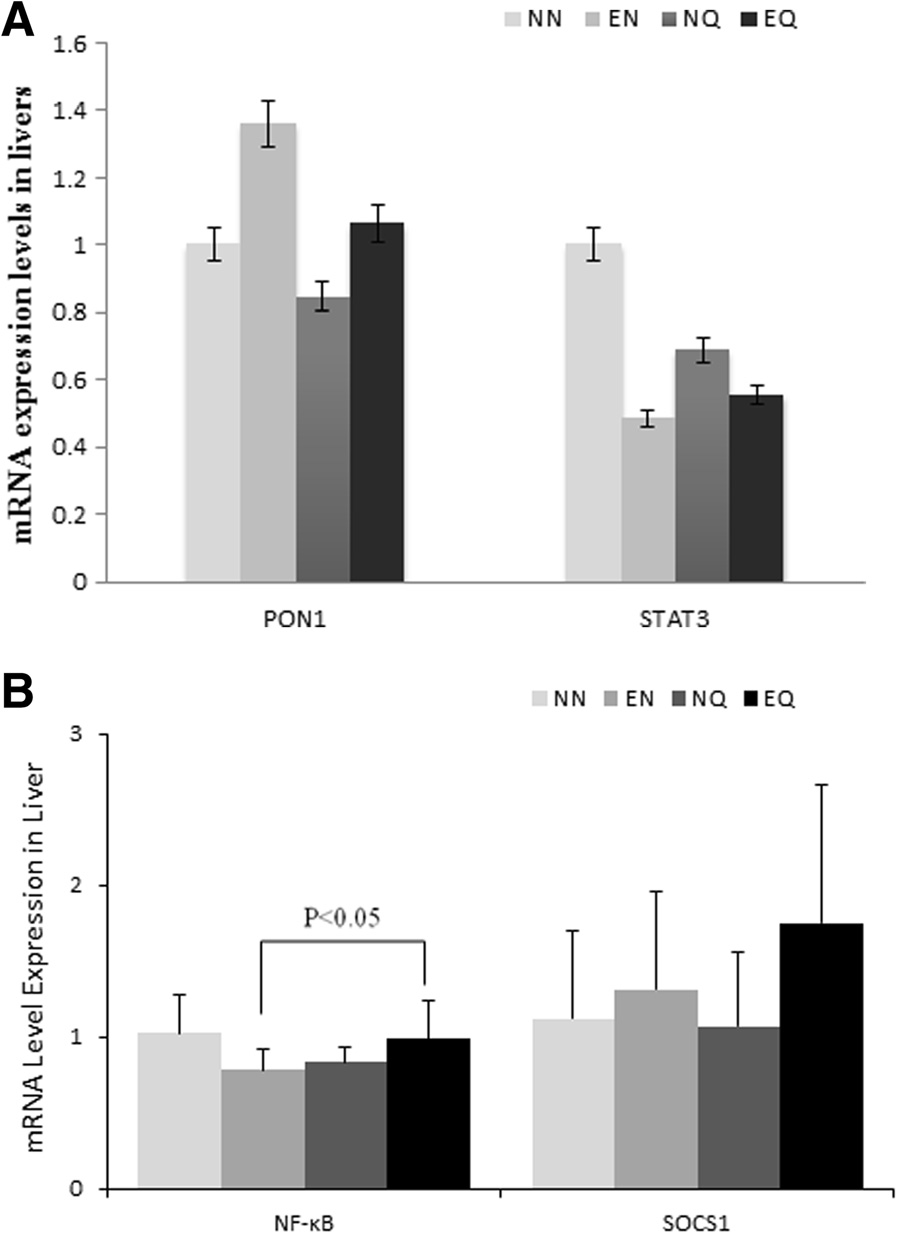
**Selected inflammation and oxidative stress associated gene expression levels.** Average relative level of mRNA expression for STAT3, and PON1 **(A)**. (The differences between the levels of PON1 and STAT3 in the various groups were not significant). **(B)** Average of relative level of mRNA of NF-κB and SOCS1expression (no significant differences between the groups), up regulation of NF-κB among the EQ is significant.

The mice in the EQ group showed a significant down regulation of apolipoprotein (APO)A-1 gene expression levels compared to NN (Figure 3A). However, the decrease in APOA-1 gene expression in the NQ and EN groups was not significantly different from the NN (Figure 3A). The APOA-1 gene expression level in the EQ group was also significantly lower (P < 0.001) compared to the EN group (Figure 3A). APOA-5 gene expression showed similar trends with all treatment groups having down regulated gene expression compared to the NN group. However, only the decrease in the EQ group was significant (P < 0.001) compared to the NN (Figure 3B). Interestingly, APOA-5 gene expression levels were significantly higher in the EQ compared to the NQ group as well (Figure 3B). Ironically, gene expressions for APOA-4, ABCA-1, and peroxisome proliferator-activated receptor (PPAR)-α followed a contrasting trend to what was observed with the APOA-1 and APOA-5. ABCA-1 gene expression was significantly (P < 0.001) up regulated in the EQ group compared to NN group (Figure 3B). Furthermore, the EQ group showed a significant (P < 0.05) ABCA-1 gene induction compared to the NQ group (Figure 3B). APOA-4 gene expression was also up regulated among all treatment groups compared to the NN group, however, only the difference between the EN and NQ groups was significant (P < 0.05) (Figure 3A). PPAR-α gene expression levels were also increased in all treatment groups compared to the NN group (Figure 3B). The EQ was shown to have the most significant induction (P < 0.001) compared to the NN group (Figure 3B). APOC-3 gene expression was up regulated with exercise, with the differences between NE group and NN group being significant (P < 0.05) (Figure 3A). A similar trend was observed between the EQ group and NQ group but not significantly (Figure 3A), which may suggest that quercetin and exercise down regulate APOC-3.The liver gene expression for the inflammatory, oxidative stress markers and transcription factors; signal transducer and activator of transcript (STAT)3, paraoxonase/arylesterase (PON)1, nuclear factor kappa-light-chain-enhancer of activated B cells (NF-κB), and suppressor of cytokine signaling (SOCS)1 showed varied responses. While exercise appears to down regulate STAT3 gene expression; it up regulated PON1 gene expression with no effect for the quercetin supplementation compared to the NN group (Figure 4A). SOCS1 was influenced by the exercise depicting up regulation in the exercise groups compared to the NN group but none of these changes was significant (Figure 4B). NF-κB gene expression levels varied, however, the EQ group had a significant (P < 0.05) induction compared to the EN group (Figure 4B).

## Discussion

Considerable attention has been given to polyphenols, such as quercetin, due to their anti-inflammatory and antioxidant properties [27-30]. Several mechanisms have been described and attributed to the anti-atherogenic effects of exercise and quercetin. It is commonly accepted that moderate exercise is an important component of a healthy lifestyle that helps to prevent or delay the onset of coronary artery disease [15-18]. These beneficial effects are lost when subjects become sedentary. Exercise intensity and duration are also critical determinants of the cardiovascular beneficial effects [32,33]. A wide range of mechanisms have been described for the beneficial effects of exercise; including: enhancing serum HDL levels; up regulation of PON1 and SRB1; inducing anti-inflammatory cytokines; and up regulation of the antioxidant enzymes contributing towards their ability to counteract the oxidative stress that is generated during exercise [34-36]. Quercetin on the other hand has been shown to act through various mechanisms mainly linked to reducing the inflammation and oxidative stress levels which are responsible for the atherosclerotic pathogenesis. Earlier studies have shown that quercetin significantly inhibit in vitro LDL oxidation, and also protects macrophages from oxidized low-density lipoprotein-induced apoptosis [37,38]. Quercetin has also been reported to inhibit the progression of atherosclerosis via up-regulating the expression of PON1 [18]; indicating a possible cholesterol reverse transportation mechanism.

Studies combining antioxidants with exercise are not new; our previous work has extensively studied the possible role of the intake of antioxidant vitamins, such as vitamin E during exercise on cardiovascular health in humans and mouse models. However, the current study is unique in a way, it has combined quercetin supplementation with exercise to examine their anti-atherogenic roles. To our knowledge this has not been explored previously. The C57BL LDLr−/− mouse model has been commonly used for the rapid development of the atherogenic diet-induced atherosclerotic plaque. One of the major findings in the current study is the atherosclerotic reduction in the three groups of mice on exercise alone, exercise with quercetin supplementation, and quercetin supplementation alone. The current quercetin dosage was selected since mice have been previously shown to tolerate and respond to this concentration (28). Exercise is well known to help reduce plaque formation [22]; however, earlier work by Parthasarathy’s group did not find significant reduction in the aortic plaque formation in exercising LDL receptor-deficient mice supplemented with vitamin E [23]. Moreover, it appears that vitamin E offset the beneficial effects of exercise by preventing the induction of aortic catalase activity and endothelial NO synthase expression [23]. The duration of this study was 30 days, which was sufficient to allow fatty streaks and plaque development to resemble early atherosclerosis development. We also chosen low intensity exercise regimen to provide the opportunity to study the effect of the combination of quercetin with low intensity exercise on the plaque formation. In the current study, we observed a 64-79% reduction in plaque formation in all treatment groups compared to control. Exercise alone greatly reduced plaque formation. Conversely quercetin supplementation alone and with exercise resulted in similar reductions of plaque formation. This outcome suggests a strong anti-athrogenic role for quercetin supplementation.

To further investigate the mechanisms that may have contributed to the reduced plaque formation, we measured plasma lipids, selected cytokines, and we assessed certain genes expression in mouse livers. Interestingly there were no significant changes in the plasma lipids profiles (data not shown). There was a slight increase in the plasma TNF-α levels in the treated groups compared to control, however, the changes between the group on the quercetin supplementation alone and the control was the only difference in TNF-α that was significant. It is not clear why this difference was observed considering the known anti-inflammatory role for quercetin. Plasma MCP-1 levels on the other hand slightly decreased with exercise or quercetin supplementation alone greatly decreased with the combination of the exercise and quercetin supplementation. MCP-1 is critical for the initiation and development of atherosclerotic lesions. It is known to participate in the progression of atherosclerosis, by promoting direct migration of inflammatory cells to the vascular wall. MCP-1 has also been detected in atherosclerotic lesions using specific antibodies [35]. It appears quercetin supplementation alone or combined with exercise has potent anti-MCP-1 effects. Plasma IL-17α levels decreased with exercise or quercetin supplementation alone and slightly increased with the combination of the two. IL-17α plays an important pro-inflammatory role in atherosclerotic plaque development. Interestingly plasma IL-17α levels were decreased with exercise or quercetin intake but not with the combination. Interleukin 17 acts as a potent mediator by increasing chemokine production in various tissues to recruit monocytes and neutrophils to the site of inflammation, its involvement in inducing and mediating pro-inflammatory responses is well understood. It is not clear if the combination of exercise and quercetin will mediate IL 17 levels as indicated by this result.

The gene expression data shown in this study for lipoprotein is differentiated. The discrepancy between the treatment and the control groups for the APOA-1, APOC-3, and APOA-5 genes cannot be explained. However, on other lipoprotein metabolism associated genes, specifically, ABCA-1, PPAR-α, and APOA-4 did show significant up regulation among the treatment groups compared to the control, indicating that quercetin supplementation alone or with exercise may modulate the reverse cholesterol transport genes. Recent reports have shown that quercetin does modulate lipid reduction. Earlier studies by us and others [19] have shown that exercise promotes plasma lipid reductions. PON1 gene expression was up regulated among exercise groups compared to the control. This data goes along the ABCA-1 data suggesting a reverse transportation mechanism which may be responsible for the decreased plaque formation. The changes in NF-κB regulations among all treatment groups compared to the control indicate a possible reduced plaque formation mechanism mediated by NF-κB. Previous studies have pointed to NF-κB as potentially one of the most important pro-inflammatory pathways in atherosclerosis [36]. NF-κB is known to be activated in smooth muscle cells, macrophages, and endothelial cells in atherosclerotic lesions. In this study its gene induction levels appears to be at the intersection of the acute inflammatory response accompanying the acute atherosclerotic plaque formation. SOCS1 and STAT3 demonstrated varied responses to exercise and quercetin supplementation between the various groups. While STAT3 gene expression levels appear down regulated in the treatment groups compared to the control, SOCS1 was up regulated in these groups compared to the control, although none of these changes were significant. SOCS-1 is known to potently restrict transduction of various inflammatory signals and, thereby modulate T-cell development. STAT3 activation by selected cytokines such IL-6 is known to preferentially induce pro-inflammatory responses, whereas other sets of cytokines such as IL-10 may activate STAT3 and promote an anti-inflammatory response. In the current study, quercetin supplementation and exercise, which are known for stimulating anti-inflammatory responses, may have activated STAT3 by a specific mechanism which resulted in decreased plaque formation [39].

In conclusion, we demonstrated that intake of quercetin alone or along with exercise will result in reduced atherosclerotic plaque formation. We speculate that these changes may have resulted from modulation of lipid metabolism, possibly by stimulating cholesterol reverse transport lipoprotein genes and through a set of anti-inflammatory cytokine genes. The manifestation of the combination of quercetin supplementation with exercise was more evident in the pro-reverse cholesterol transport genes indicating a plausible mechanism for their beneficial effect.

## Competing interests

The authors declare that they have no competing interests.

## Authors’ contributions

All authors read and approved the final manuscript.
